# A High-Density Microchamber Array for the Analysis of Extracellular Vesicles Derived from Single Cells under Drug Treatment

**DOI:** 10.1021/acs.analchem.5c05621

**Published:** 2026-01-05

**Authors:** Lucien R. Stöcklin, Claudius L. Dietsche, Petra S. Dittrich

**Affiliations:** 27219 ETH Zürich, Department Biosystems Science and Engineering, CH-4056 Basel, Switzerland

## Abstract

Extracellular vesicles (EVs) are key players in cancer development and drug resistance. For example, heat shock protein 90 (HSP90) carried via EVs from secreting cancer cells to distant recipient cells mediates apoptosis and metastasis. Here, we study EV secretion from individual breast cancer cells and the changes under treatment with the HSP90-inhibiting cancer drug tanespimycin (17AAG). We introduce a two-layer microfluidic platform with an array of microchambers that coencapsulate single cancer cells with functionalized beads for the capturing and immunostaining of EVs. Microchambers are created by pressurizing a microfluidic layer with densely packed, round openings, which are aligned with cell- and bead-traps. This new design facilitates the isolation of cells in over 5100 microchambers and efficient EV capture. We characterize the EV secretion of two breast cancer cell lines: triple-negative MDA-MB-231 cells and HER2-positive SkBr3 cells secrete EVs that carry distinguishable levels of tetraspanins (CD9, CD63, and CD81) and HSPs (−90 and −70). Upon drug treatment, the signals for HSP90- and HSP70-positive EVs increase for both cell lines. However, analysis of protein colocalization on the EV surface revealed a significant difference in EV subpopulations: while MDA-MB-231 cells have no HSP90 on CD63-positive EVs, these two markers are colocalized on SkBr3-derived EVs, indicating different intracellular biogenesis pathways for HSP90-loaded EVs. Moreover, our results emphasize that using CD63 as the sole EV capture protein may hide important EV subpopulations. Overall, our platform may support future choices of EV biomarkers for diagnostic and biomedical purposes and help in understanding the heterogeneous drug response of cancer cells.

## Graphical abstract

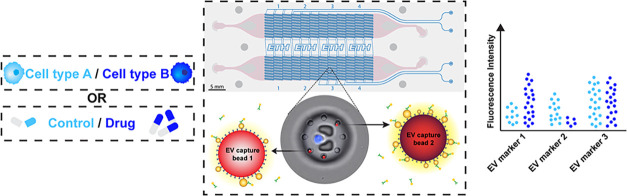

## Introduction

Extracellular vesicles (EVs) play a major role in the development and treatment of breast cancer.^1^ These nanometer-scale spherical particles are secreted from parental cells and carry messengers to surrounding recipient cells, opening the possibility of a highly specific cell-to-cell communication.^2^ Various studies have demonstrated the involvement of EVs in the formation of metastasis,^3^ vascularization of the tumor,^4^ modulation of the immune response,^5^ as well as in the resistance to cancer drugs.^6^ A broad range of membrane-bound proteins are found on the surfaces of EVs. The exact composition of EVs is largely determined by their biogenesis pathway.^7^ Common proteins include tetraspanins such as CD63, CD9, or CD81, heat shock proteins (HSPs) (mainly HSP90 and HSP70), and epithelial cell adhesion molecule (EpCAM).^8^ While these membrane markers are primarily crucial for the adhesion of EVs to their target cell, they can also initiate specific intracellular responses in the recipient cells.^9^ In addition, some studies have revealed altered levels of these proteins on the EV membrane in various forms of cancers.^10–13^ For example, EV-bound HSP90 is largely involved in the communication between breast cancer cells and the surrounding connective tissue.^14^ Therefore, there is a significant interest in profiling these EV surface markers for diagnostic purposes.^2^

The well-known intercellular heterogeneity within a solid tumor^15^ extends to the level of secreted EVs.^16,17^ Indeed, heterogeneity is not only found among EVs released by distinct parental cells but also within individual cells, which release diverse EV subtypes.^13^ Conventional methods for the analysis of cancer-derived EVs rely on bulk measurements, and typically result in time-consuming, costly, and prone-to-bias approaches.^18^ Moreover, they generally do not allow to resolve the different EV subpopulations.^19^ Instead, microfluidic platforms offering single-cell resolution have been proposed.^20^ Individual cells are usually isolated inside microcompartments and secreted EVs commonly captured by affinity. Optical detection of immobilized EVs can then be achieved using a sandwich immunoassay with fluorescent reporters.^21–25^ However, the need to prefunctionalize the devices with the capture antibodies results in long preparation times, lower versatility, and restricted number of capture antibodies which can be used simultaneously. In addition, the currently existing platforms do not enable to quantitatively profile different EV subpopulations secreted at the single-cell level, and the multiplexing capabilities of these technologies remain limited by the spectral overlap of the fluorescent markers.^26^

Here, we present a novel microfluidic platform which enables the multiplexed and semiquantitative profiling of single-cell-derived EVs. Individual cells are entrapped within a high-density array of pressure-controlled microchambers. Up to eight conditions can be achieved on a single device with over 5100 microchambers in total. Secreted EVs are then immobilized on magnetic beads functionalized with a capture antibody. Multiplexing capabilities, image acquisition, and sensitivity are simultaneously improved by trapping beads at defined positions and utilizing fluorescently barcoded magnetic beads in combination with a single fluorescent reporter for the detection of all EV phenotypes. We study EVs secreted from two breast cancer cell lines, triple-negative MDA-MB-231 and HER2-postive SkBr3. Both cell lines are characterized by no expression of estrogen and progesterone receptors (ER and PR, respectively).^27^ The absence of these endocrine receptors results in aggressive phenotypes often associated with a poor prognosis and the inability to treat patients with conventional therapies which target hormonal receptors.^28^ Tanespimycin (17AAG) is such a drug that does not bind to hormonal receptors but inhibits HSP90, leading to incomplete folding and degradation of HSP90-client proteins and, finally, to apoptosis of the cancer cells. 17AAG has been effectively used to treat various kinds of solid tumors,^29^ including HER2-positive breast cancers^30^ and, more recently, also triple-negative breast cancer.^31,32^ As HSP90 is secreted via EVs from the cells, we investigate here, among others, the presence of HSP90 on EVs from treated and untreated cells. Our overall goal is to determine how EV secretion patterns differ in a population of cancer cells across cell lines and how drug treatment affects these patterns.

## Experimental Section

### Operation of the Microfluidic Device

Fabrication and preparation of the device are described in the Supporting Information. Magnetic beads functionalized with the different EV capture antibodies (ProcartaPlex) were flushed inside the flow-controlled channels. A pipet tip loaded with the magnetic bead mix was plugged at the outlet of each channel, and the bead mix was sucked in at a −3 μL/min flow rate. When the beads reached the channel inlet, the negative flow was stopped and the magnet array was placed on top of the device. A positive flow rate of 0.5 μL/min was then applied to capture the beads inside the magnetic bead traps. Nontrapped beads were washed away at 20 μL/min for 3 min. Next, cells were loaded onto the chip following the same principle. A pipet tip loaded with the cell suspensions (0.8 million cells/mL) was plugged at the outlet of each flow-controlled channel and captured by the hydrodynamic traps at a positive flow rate of 1 μL/mL. When most of the cell traps were occupied, a pressure of 1500–1800 mbars was applied in the pressure-controlled channels, resulting in the closing of the ring-shaped valves and consequently isolating each cell from each other. The remaining nontrapped cells were washed away at 10 μL/min for 3 min. During the incubation of 4 h at 37 °C with 5% CO_2_ and >85% humidity, microscopy images of the cells were acquired to register the microchambers which contained one live cell. After incubation, the pressure in the pressure-controlled channels was slowly released and a 20 μL/min flow rate was applied in the flow-controlled channels to wash the cells away. Next, the DMEM syringes were exchanged with syringes filled with wash buffer to conduct the sandwich immunoassay and prevent nonspecific binding on the beads’ surface. The detection antibody mixture, specified in the result section for every experiment, and streptavidin–phycoerythrin (SA-PE) solution were sequentially flushed in at −3 μL/min, incubated for 30 min at room temperature, and washed away at 20 μL/min for 3 min. Finally, reading buffer was flushed in and fluorescence images of the magnetic beads were acquired.

### Image Acquisition

Images were acquired on a Nikon Ti2-E inverted epifluorescence microscope mounted with a DS-Qi2 digital camera. For sensitive measurements of EVs on magnetic beads, a 20× objective with a numerical aperture of 0.75 was used (Plan Apochromat 20×), and for imaging of cell viability on the microfluidic device, a 10× objective with a numerical aperture of 0.45 was used (Plan Apochromat 10×). The fluorescently barcoded magnetic beads were imaged with two separate filter cubes in the far-red spectrum region: one with 628/670 nm excitation/emission (ex/em) and the other with 640/711 nm ex/em. The PE readout of the sandwich immunoassay was measured using a 532/575 nm ex/em filter cube and the live staining of the cells with a DAPI filter cube (359/457 nm ex/em).

### Data Processing and Statistical Analysis

Collected data was processed with in-house developed MATLAB-based software.^33^ The image processing pipeline is detailed in the Supporting Information (Figure S1). Data was then analyzed using Python (version 3.10.5). Statistical tests included Levene’s tests to assess equality of variance, followed by independent Student *t* tests or Welch’s tests (Figures 3B, 4B,C, and 5B–E). *p*-Values below 0.01 were treated as significantly different (*p*-values below 0.01, 0.001, and 0.0001 are indicated by *, **, and *** respectively).

## Results and Discussion

### Performance of the Microfluidic Platform

The microfluidic device has two layers made of poly­(dimethylsiloxane) (PDMS, Figure 1). The upper layer comprises two parallel (and identical) flow-controlled channels where single cells are hydrodynamically trapped. Underneath, a thinner pressure-controlled layer enables the on-demand actuation of four independent valves and the isolation of single cells inside microchambers with a volume of 240 pL. The compact arrangement of the ring-shaped valves allows to reach a density of 5184 microchambers per device, which corresponds to a 4-fold increase compared to previously established valve-based microchamber systems.^21,22,33–35^ Furthermore, separating both flow-controlled channels in four isolated valve compartments allows one to run up to eight different conditions on a single device. Within each microchamber, eight microwells for capturing antibody-functionalized magnetic beads are located. After affinity capture of EVs on the beads’ surface, specific detection antibodies were supplied for immunostaining of target proteins. Each bead carries a fluorescent barcode to identify the capture antibody immobilized on a specific bead (Figure S2, Table S1). Another fluorophore, PE, was used as a tag for all detection antibodies, maintaining image acquisition to only three fluorescence channels independent of the number of EV markers analyzed. The quantification and multiplexing capabilities of the platform were validated with calibration curves of protein standards (Figure S3) and enriched EV samples (Figure S4). In addition, the specificity of EV capture on the functionalized magnetic beads was validated with scanning electron microscopy (SEM) measurements from enriched EV samples (Figure S5).

**Figure 1:**
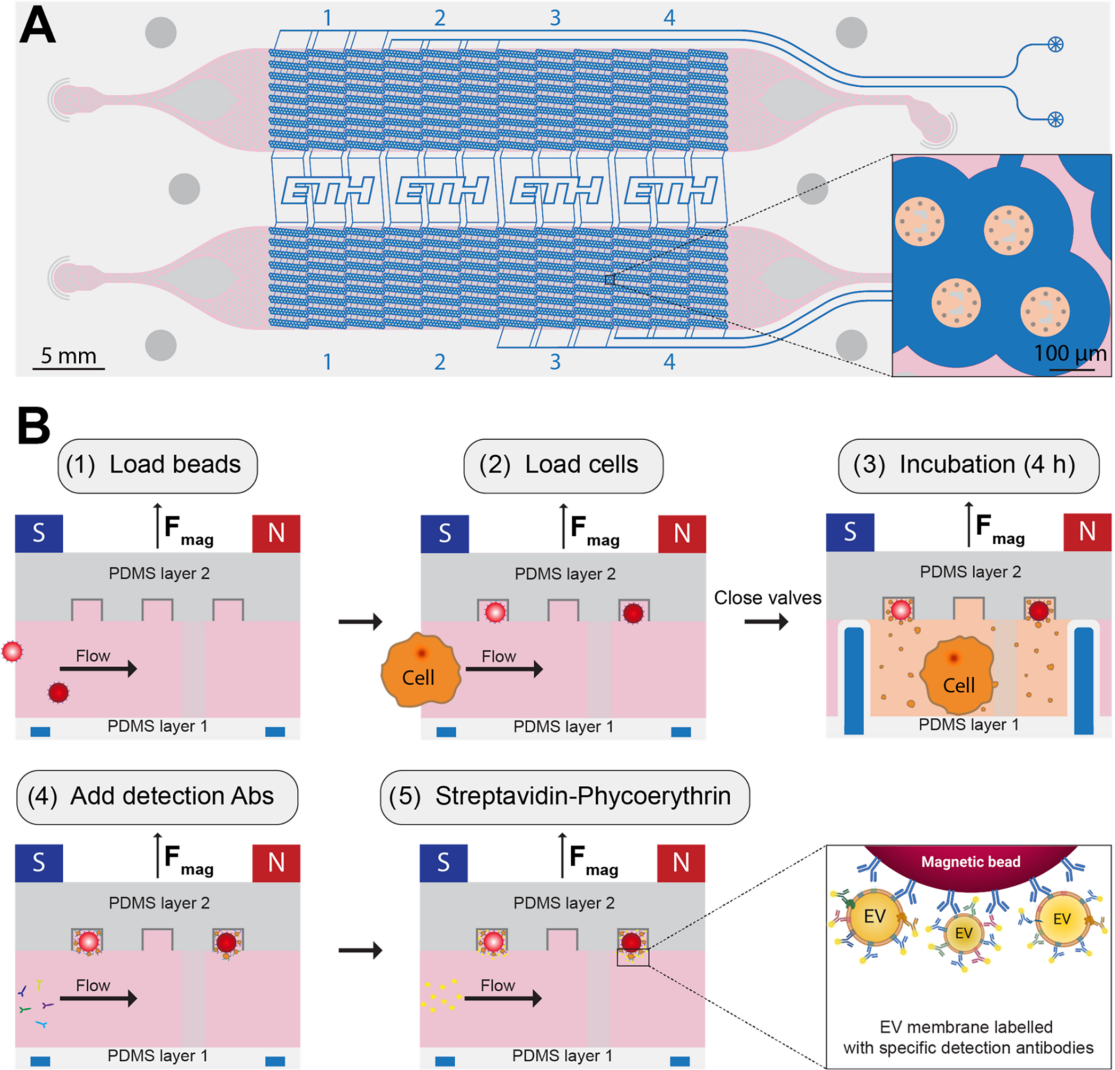
Design and operation of the microfluidic platform. (A) Top view of the two-layer PDMS device. The four independent pressure-controlled valves (blue) are aligned underneath two parallel flow-controlled channels (pink), allowing to run up to eight conditions on a single device. The six darker gray circles are indication marks to place the magnet array on top of the chip. The magnified view of four microchambers shows two pillars (light gray), which are used for the hydrodynamic trapping of individual cells and eight microwells for trapping the functionalized magnetic beads (dark gray). (B) Side view of one microchamber during the 5 experimental steps of the assay.

We first characterized bead and cell capture efficiencies. Over 80% of the microchambers contained at least one magnetic bead. More importantly, over 50% of all microchambers contained two or more beads, critical for downstream multiplexing analyses (Figure 2A). The robustness of bead capture during device operation was also confirmed (Figure S6). Single-cell capture reached 48%, with around 21% of the microchambers remaining empty (Figure 2B). Figure 2C shows a typical microscopy image of a microchamber where an individual MDA-MB-231 breast cancer cell was successfully cocaptured with three magnetic beads. Next, to assess whether sealing of the microchambers upon closing of the pressure-controlled valves remained stable for a prolonged period, an incubation experiment with fluorescent dyes was performed. After more than 5 h of incubation, the fluorescein solution was still retained inside the microchambers, indicating no leakage from the microchambers (Figure 2D). This was further confirmed in the on-chip cell experiments, where the overall fluorescence signal measured on magnetic beads inside empty microchambers was significantly lower than the signal measured within cell-containing microchambers (Figure S7).

**Figure 2:**
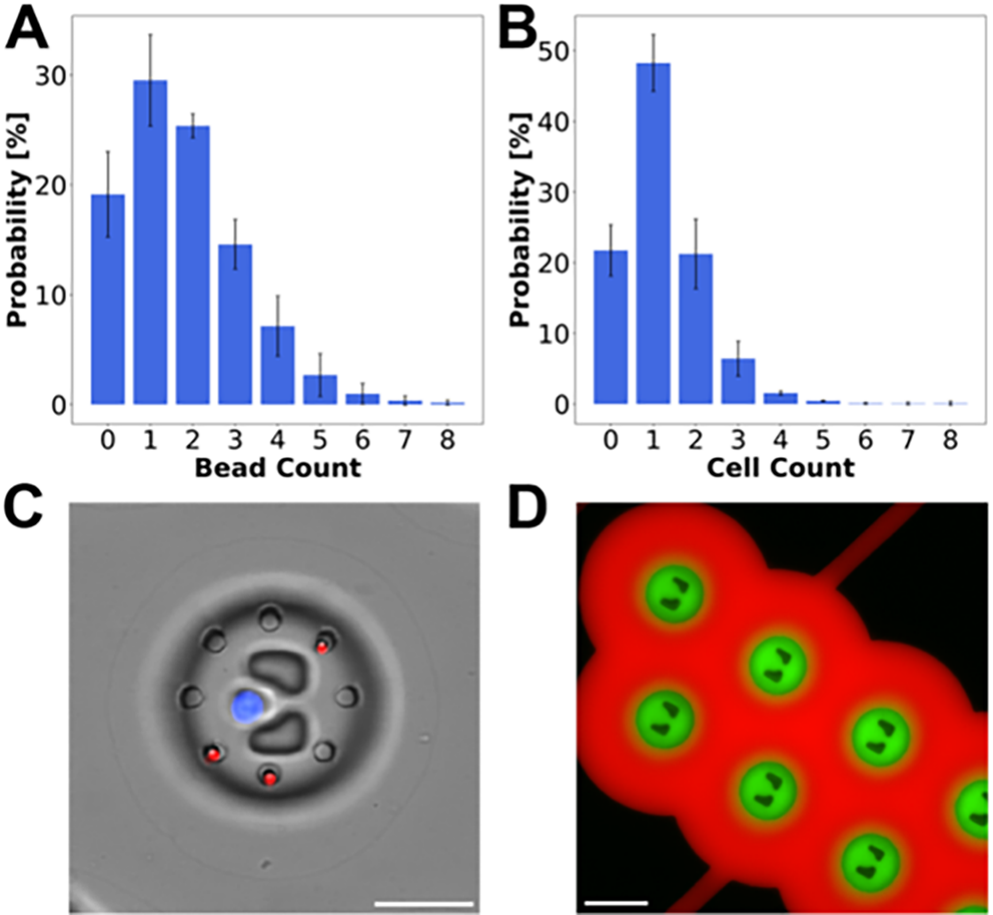
Characterization and performance assessment of the microfluidic device. Histograms of bead (A) and cell (B) distribution. (C) Representative brightfield microscopy image of one microchamber with a trapped cell stained with Calcein Violet AM (blue) and three fluorescently barcoded magnetic beads (red). Scale bar = 50 μm. (D) Epifluorescence microscopy image recorded more than 5 h after filling, showing the stable sealing of the microchambers. Flow-controlled channels were filled with 10 μM fluorescein (green) and the pressure-controlled channels were filled with 10 μM sulforhodamine B (red). Scale bar = 100 μm.

### Profiling Single-Cell-Derived EVs from Different Breast Cancer Cell Types

Next, we investigated EVs secreted from two breast cancer cell lines, MDA-MB-231 and SkBr3 cells. The cells were incubated inside the closed microchambers for 4 h, together with functionalized barcoded beads targeting common EV membrane proteins (tetraspanins CD63 and CD81, HSP70, HSP90, and EpCAM).^10,36,37^ The incubation time was based on prior evidence that EVs are mainly secreted in this time range.^38^ The distribution of beads is stochastic but allows us to obtain information about all of the different proteins in a single experiment. As more than one bead can be located in one chamber, we confirmed that there is no affinity competition between different capture antibodies and the secreted EVs (Figure S8). In addition, we use a mixture of detection antibodies, all with the same fluorescent tag (PE), to label tetraspanins CD63 and CD81, EpCAM, and the heat shock proteins on the EV surface at the same time (Figure 3A), similar to a previous study.^39^ In contrast to the commonly employed strategy to label one selected membrane protein (mainly CD63),^23,24,40^ our approach maximizes the fluorescence signal and labels more EVs, i.e., increases the sensitivity.

**Figure 3:**
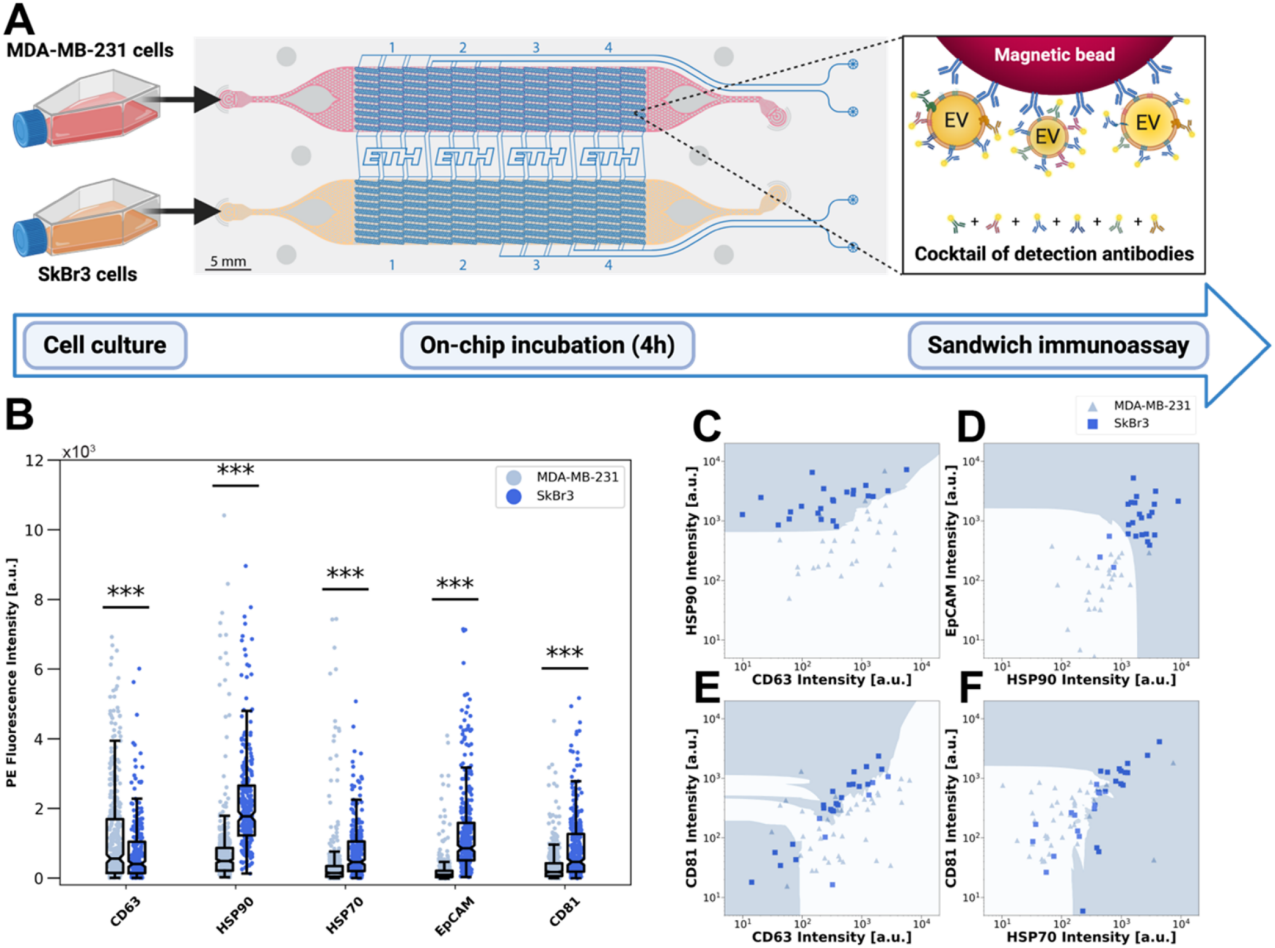
Profiling single-cell-derived EVs from two different breast cancer cell types, MDA-MB-231 and SkBr3. (A) Schematic of the experimental procedure. Magnetic beads are functionalized with antibodies targeting one of five distinct EV surface markers; detection is based on a cocktail of five PE-labeled detection antibodies (CD63, HSP90, HSP70, EpCAM, CD81). (B) Fluorescence intensity measured on magnetic beads; each data point represents a single cell. For each bead type and each cell type, *n* ≥ 278 (*N* = 2). (C–F) Pairwise plots for four of the ten EV marker pairs. Decision boundaries obtained with the KNN classifier are displayed. For each bead pair and each cell line, *n* ≥ 25. Image A was created in BioRender. Stocklin, L. (2025) https://BioRender.com/u8x2fn8.

Figure 3B shows the results for the two cell lines, each for the five different EV markers. We observe a high intercellular heterogeneity, which is in accordance with previous studies.^17^ Significant differences can be seen for the two cell types. We detect higher signals for CD63-positive EVs derived from MDA-MB-231 cells, while a higher fluorescence was measured for HSP90-, HSP70-, CD81- and EpCAM-positive EVs secreted by SkBr3 cells. Low signals for EpCAM-positive EVs were expected for mesenchymal cells such as MDA-MB-231 compared to epithelial cells such as SkBr3 cells,^41,42^ which reflects the different expression of EpCAM in both cell lines (Figure S9). Interestingly, a small proportion of MDA-MB-231 cells showed much higher signals from HSP70- and HSP90-positive EVs, despite their average lower levels. A bulk analysis of cell-derived EV secretions would not enable detection of such outliers as the higher signals would be hidden within the population mean.

Despite the different patterns, the considerable overlap between the two cell lines in Figure 3B hinders the ability to determine the origin of the EVs from a single parameter. Since many microchambers hosted two distinct EV capture beads, a pairwise analysis can be achieved (Figure 3C–E and complete overview in Figure S10). A nonlinear k-nearest neighbor (KNN) classification algorithm was applied to define decision boundaries for classification, as shown with the colored areas on each pairwise plot. Overall, studying the paired signals of EV secretions enabled clearer separations between the two cell lines. SkBr3 cells secreted HSP90- and EpCAM-positive EVs that led to significantly higher fluorescence signals compared to those of MDA-MB-231 cells. Incorporating these EV markers into the analysis therefore resulted in the most optimal classification performances (accuracy scores above 90%, see Table S2), highlighting that both proteins could be critical EV membrane markers for the classification of SkBr3 and MDA-MB-231 cells^10,43–45^ (Figure 3C,D). On the contrary, the pairwise analysis of CD63- with CD81-positive EVs resulted in a lower accuracy score (80%) and an inconclusive classification (Figure 3E), in accordance with well-known observations that these tetraspanins are heterogeneously expressed on EV membranes.^46,47^ Among all marker pairs, CD81 and HSP70 yielded the lowest classification accuracy (73%, Figure 3F).

Last, the correlation maps summarize cosecretion of EV phenotypes from the same cell (Figure S11). It may identify EV subpopulations which share common biogenesis or secretion pathways, e.g., correlation between CD63- and HSP90-positive EVs observed with SkBr3 cells implies a distinct secretion mechanism compared to MDA-MB-231 cells. It should be noted that the correlation does not reveal the colocalization of the selected markers on the *same* EV, which is discussed below (Figure 5).

In summary, the analysis allowed us to select marker pairs that could be used, e.g., in liquid biopsies to differentiate these similar breast cancer cells.

### Changes in Single-Cell-Derived EV Secretion upon 17AAG Drug Treatment

In the next experimental series, we investigated whether treatment with the HSP90-inhibiting drug 17AAG influenced EV secretion and protein composition of the EV membrane. Cells were treated for 24 h with 10 μM 17AAG prior to loading on one channel of the microfluidic device (Figures S12,S13). A control sample (treatment with dimethyl sulfoxide, DMSO) was loaded in the second channel of the device. We used the same EV capture and detection procedure as before, here with beads targeting either one of the three tetraspanins (CD9, CD63, and CD81), or one of the two heat shock proteins (HSP90 and HSP70). For all experiments, bead-captured EVs were labeled with a mixture of detection antibodies targeting the three tetraspanins and the heat shock proteins (Figure 4A).

For both cell lines, we found a significant increase in the fluorescence signals for EVs that were captured on the heat shock protein-targeting beads (Figure 4B,C, (i)) compared to the control. The allosteric binding of 17AAG to the N-domain of chaperone HSP90 directly impedes the folding of its client proteins and results in their degradation.^30,31^ In response, the cancer cells increase the production and secretion of HSPs, including HSP70 via an induction mechanism.^48^ Moreover, the upregulation of HSPs has been observed at the EV level.^32^ The increase in the HSP-related signal was therefore expected. In addition, MDA-MB-231 cells show slightly higher signals for the tetraspanin-captured EVs (mainly CD63-captured EVs), which could be attributed to the larger amount of EVs that are secreted by the 17AAG-treated MDA-MB-231 cells, as confirmed by nanoparticle tracking analysis (NTA) (Figure S14A). However, if such signal increase is solely due to a higher number of secreted EVs, or also the result of higher protein expression on the EV membrane, it cannot be resolved at this stage. In contrast, lower signals were measured for CD63-captured EVs from drug-treated SkBr3 cells.

Next, a pairwise analysis from microchambers which contained two distinct EV capture beads is done (Figures 4B,C, ii-iv, S15,S16). Shifts to the upper right corner indicate a higher release of EVs positive for these respective markers and/or a higher protein expression on the EV surface. Again, for both cell lines, strong upward shifts in fluorescence signals can be observed for pairs involving HSPs, while CD63-captured EVs are only upshifted for MDA-MB-231 cells. These results suggest a distinct drug response of MDA-MB-231 cells compared to SkBr3 for tetraspanin-captured EVs, and the modulation of cell-type-specific EV biogenesis pathways.

**Figure 4:**
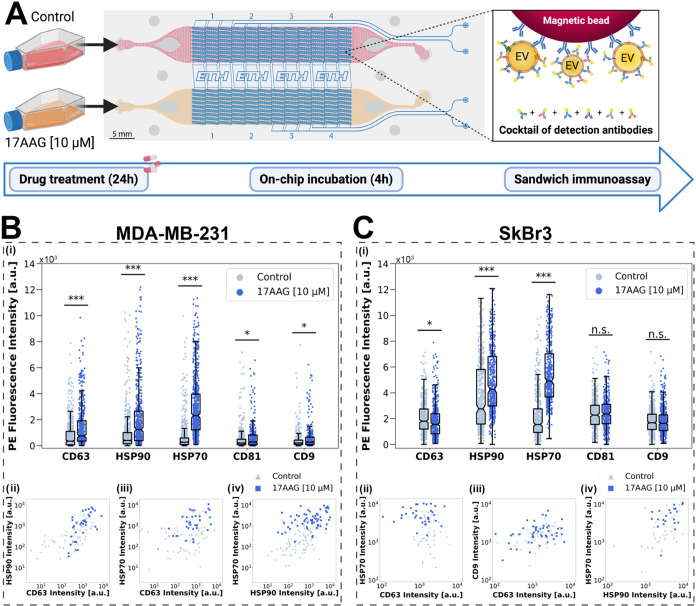
Effect of 17AAG treatment on EV secretion. (A) Schematic of the experimental procedure. Treated and control cells were detached, resuspended, and loaded on-chip. Following a 4- h incubation, EVs captured on the magnetic beads were labeled with a cocktail of detection antibodies sharing PE as a common fluorescent reporter (CD63, HSP90, HSP70, CD81, and CD9). Two breast cancer cell lines, (B) MDA-MB-231 and (C) SkBr3, were investigated. (i) PE fluorescence intensity measured on magnetic beads functionalized with antibodies targeting one of five different EV surface markers (CD63, HSP90, HSP70, CD81, or CD9). For each bead type and each condition, *n* ≥ 301 (*N* = 3). (ii–iv) Pairwise plots for three of the ten EV marker pairs (complete pairwise plots are shown in Figures S15,S16). Drug-treated and control conditions are displayed and compared. For each bead pair and each condition, *n* ≥ 25. Image A created in BioRender. Stocklin, L. (2025) https://BioRender.com/o05c901.

### Proteins Colocalization on the EV Surface under 17AAG Treatment

Next, we employed our approach to further investigate protein colocalization on the EV surface. In these experiments, we used CD63 to capture the secreted EVs as a widely accepted, common marker.^23–25,40^ The four independent pressure-controlled regions in the microfluidic channels were leveraged to stain the captured EVs with a single detection antibody per region (Figure 5A). Again, cancer cell lines MDA-MB-231 and SkBr3 were studied. Controls with a healthy breast cell line, MCF10A, are depicted in Figures S17,S18 and show a similar response to the drug as SkBr3 cells–both epithelial.

Figure 5B depicts the single-cell results obtained from the MDA-MB-231 cells. Higher signals are measured for the tetraspanin-positive EVs.^25^ In particular, we can confirm the colocalization of CD63 and CD9 on EVs as reported in a prior study.^49^ Signals for the heat shock proteins are low and remain low after drug treatment, indicating that they are not colocalized with CD63. MDA-MB-231 cells secrete EVs with HSPs as seen in Figure 4B, but these EVs may be formed by an unknown biogenesis pathway, as speculated by Tang et al.^14^

Analysis of the SkBr3 cells (Figure 5C) shows again the colocalization of CD63 and CD9 on the EV membrane. The presence of EpCAM is confirmed on CD63^+^-EVs as well. We found a remarkably large signal when the captured EVs were stained for HSP90, which was further increased upon treatment with 10 μM 17AAG. As the number of secreted EVs did not increase upon drug treatment (NTA measurements, Figure S14B), we hypothesize that EVs carry more HSP90 on their surface in response to the drug. In contrast, tetraspanins- and EpCAM-positive EVs decreased after the treatment. Next, a dose–response analysis of SkBr3 cells enabled to estimate the concentration thresholds at which a measurable change in specific EV subpopulations occurs. Following a 24 h treatment with varying 17AAG concentrations (0–50 μM), individual SkBr3 cells were incubated and CD63^+^ EVs detected with antibodies against CD9 or HSP90 (Figure 5D,E). A significant drop in the fluorescence signal from CD63^+^-CD9^+^ EVs is observed above 10 μM 17AAG, while the signal for CD63^+^-HSP90^+^ EVs already increased at 1 μM.

**Figure 5:**
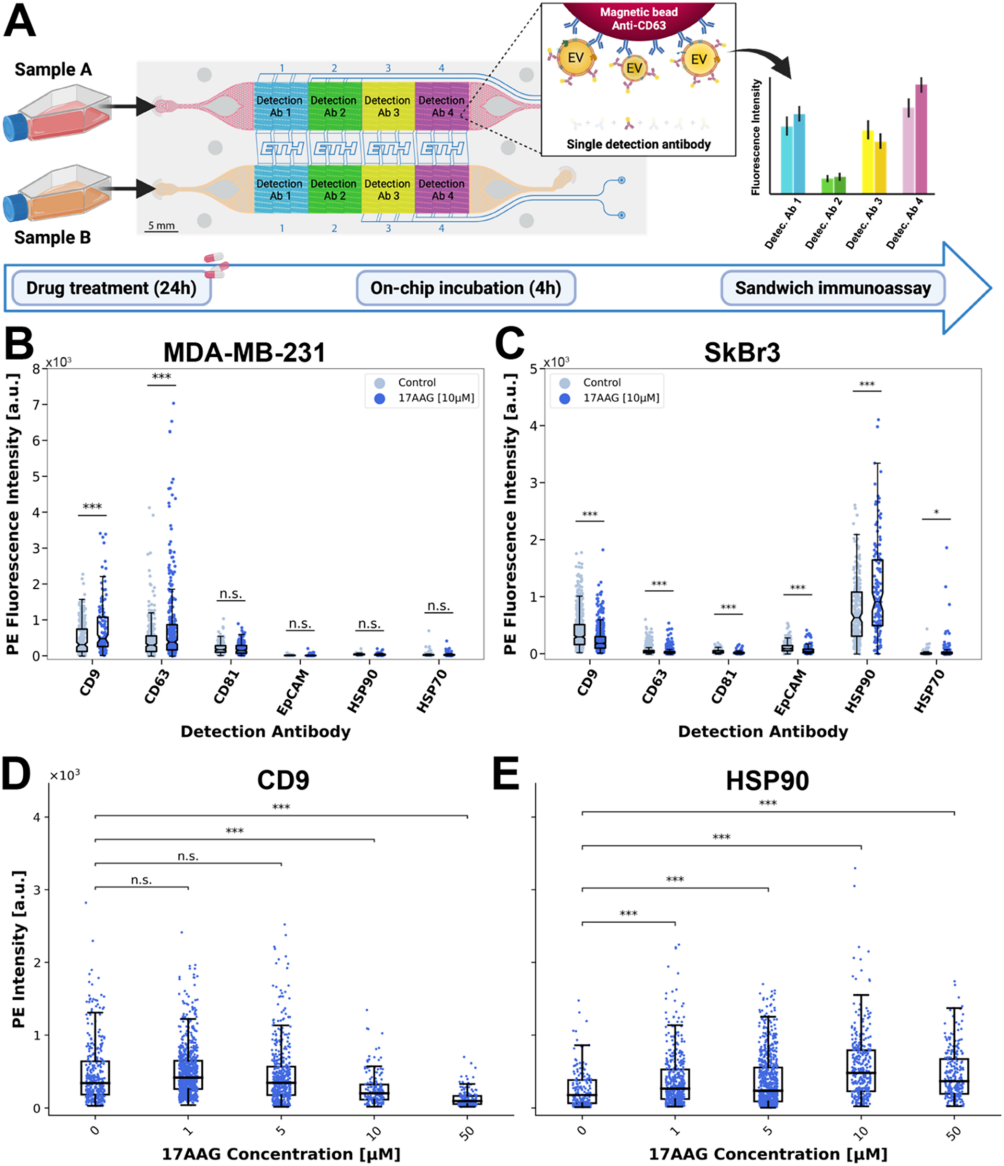
Colocalization of proteins on CD63-positive EVs, derived from single cells. (A) Schematic representation of the workflow. Cell suspension was introduced, and CD63-positive EVs were captured on magnetic beads. The valves were used to open subsets of chambers and introduce one of six different detection antibodies. EVs coexpressing CD63 and the surface protein corresponding to the detection antibody employed were thus selectively labeled. (B, C) PE fluorescence intensity measured on the magnetic beads, after EV capture and subsequent fluorescent labeling with the different detection antibodies for (B) MDA-MB-231 and (C) SkBr3 cell lines. For each cell type and each labeling condition, *n* ≥ 108 (*N* = 2). (D-E) Drug–dosage response from individual SkBr3 cells at increasing concentrations of 17AAG (0–50 μM). Secreted EVs were detected with detection antibodies against (D) CD9 and (E) HSP90 (*n* ≥ 166, *N* = 1). Image A was created in BioRender. Stocklin, L. (2025) https://BioRender.com/al7a4xx.

The disparities in protein colocalization observed between SkBr3 and MDA-MB-231 cells unveil strong and formerly unknown differences in EV biogenesis pathways, which can also influence the overall response of the cells to the drug. In addition, the increased fluorescence signal for tetraspanin-positive EVs secreted by MDA-MB-231 cells may indicate the activation of EV-mediated drug-resisting pathways specifically in triple-negative cancer subtypes. In contrast, the decreased signals of tetraspanin-positive EVs secreted by SkBr3 cells suggest that no rerouting via tetraspanin-positive EVs occurs in the HER2-positive cell type upon 17AAG treatment^30^ and could explain the higher sensitivity of SkBr3 cells to 17AAG.^50^ Together, our results demonstrate that the effect of 17AAG extends beyond the upregulation of HSP-positive EVs reported so far^32,51^ and advocate for further research in this direction.

## Conclusion

Profiling of EVs released by individual cells aims at unraveling the inherent heterogeneity of secreted vesicles. Here, we propose a microfluidic platform designed for the multiplexed analysis of single-cell-derived EVs. Our system has successfully profiled and classified two distinct breast cancer cell lines. By selecting critical EV membrane markers (here EpCAM and HSP90) and using a k-nearest neighbor classifier, we can reliably link EVs to their parental cell. The marker pairs could serve as targets, when EVs are used as biomarkers, e.g., in liquid biopsies. Moreover, the EV protein profiles also indicate the effect of drug treatment and hence can be employed for monitoring the effect of drug treatment; e.g., susceptible cells change the EV protein profile. Here, we investigated how 17AAG treatment influences the EV production of MDA-MB-231 and SkBr3 cells and found significantly increased signals for EVs carrying heat shock proteins HSP90 and HSP70. As the drug prevents correct folding of the HSPs, the cells produce more HSPs and transport the protein out via EVs. Remarkably, this transport is different for SkBr3 and MDA-MB-231. SkBr3 cells secrete a unique subtype of EVs coexpressing CD63 and HSP90, for which the fluorescence signal increased under drug treatment, while that of another phenotype positive for two tetraspanins, CD9^+^-CD63^+^ EVs, dropped. In contrast, the treatment with 17AAG resulted in an increased signal for CD9^+^-CD63^+^ EVs in MDA-MB-231 cells, while almost no CD63^+^-HSP90^+^ EVs were detected. We therefore can conclude that the two breast cancer cell lines use different strategies in response to drug treatment. Notably, SkBr3 cells are more sensitive to 17AAG and may react more strongly than MDA-MB-231 cells at the given drug concentration.^50^ Our platform has also enabled the estimation of the critical drug dose at which specific EV subpopulations are affected. These observations highlight the activation and regulation of distinct EV secretion pathways across different subtypes of breast cancers, which are needed to understand the EVs’ role in tumor development. In the future, the studies could be expanded to monitor drug resistances mediated by EVs.

In summary, the versatile use of our microfluidic platform revealed new pieces of the puzzle of EV secretion from single cancer cells and can therefore contribute to understanding the complex biogenesis and secretion pathways of EVs from single cells. Given the potential use of EVs as biomarker carriers and for treatment monitoring, the current method advises on the choice of surface markers that may reveal the clearest differentiation between different cell types and under different drug treatments.

## Supplementary Material



## Notes

Image processing workflow; characterization experiments; complete pairwise data; and additional methods (Figures S1–S18, Tables 1–3) (PDF)
