# Supplementary material for: A High-Density Microchamber Array for the Analysis of Extracellular Vesicles Derived from Single Cells under Drug Treatment
Source: Anal Chem. 2026 Jan 5;98(2):1460–9. doi: 10.1021/acs.analchem.5c05621 (PMC12824987; doi:10.1021/acs.analchem.5c05621)
Supplement: Supplementary file 1 [file ac5c05621_si_001.pdf]

Supporting Information for

A high-density microchamber array for the analysis of extracellular vesicles derived from single cells under drug treatment

*Lucien R. Stöcklin, Claudius L. Dietsche, Petra S. Dittrich**

ETH Zürich, Department Biosystems Science and Engineering, CH-4056 Basel, Switzerland

*corresponding author
e-mail: petra.dittrich@bsse.ethz.ch

Table of Contents

List of figures ***2***

List of tables ***2***

Experimental section ***16***

Microfabrication **16**

Assembling of the microfluidic device **16**

Preparation of the microfluidic device **16**

Cell culture **17**

EV enrichment from cell culture media **17**

Scanning electron microscopy (SEM) of bead-bound EVs **17**

References ***18***

List of figures

Figure S1. Image acquisition and processing pipeline of the on-chip single-cell experiments.
Figure S2. Classification of the different bead types based on two fluorescent barcodes.
Figure S3. Calibration curves obtained on a single device for the six protein standards of interest.
Figure S4. Calibration curve of enriched EV samples obtained on a single device.
Figure S5. Scanning electron microscopy (SEM) image of bead-bound EVs.
Figure S6. Bead stability inside the microchambers after the different steps of device operation.
Figure S7. Comparison of the output PE fluorescent signal on magnetic bead localised in chambers which contained no cell with microchambers which contained one or more cell(s).
Figure S8. Comparing fluorescent signals in microchambers which contained a single bead with microchambers which contained two or more beads.
Figure S9. Brightfield images of MDA-MB-231 and SkBr3 cells in 2D cell culture.
Figure S10. Complete pairwise plots for all ten EV marker pairs.
Figure S11. Correlation maps for all ten EV marker pairs for the two cell lines of interest
Figure S12. Brightfield images of MDA-MB-231 and SkBr3 cells during the drug treatment assay.
Figure S13. Cell viability assay upon treatment with different 17AAG concentrations.
Figure S14. Nanoparticle Tracking Analysis (NTA) results of EV samples enriched from bulk cell culture media.
Figure S15. Complete pairwise plots for all ten EV marker pairs for the 17AAG drug treatment experiment with MDA-MB-231 cells.
Figure S16. Complete pairwise plots for all ten EV marker pairs for the 17AAG drug treatment experiment with SkBr3 cells.
Figure S17. Colocalization of proteins on CD63-positive EVs, derived from single MCF10A cells.
Figure S18. Effect of 17AAG on EVs secreted by healthy breast cell line MCF10A.

List of tables

Table S1. Catalogue numbers of the capture (bead-bound) and detection (in solution) antibody kits (ProcartaPlex^{TM}).
Table S2. Accuracy score of the KNN classifier for each EV marker pair.
Table S3. Summary of the antibodies used for capturing (bead-bound) and detecting (in solution) the single-cell-secreted EVs in the different experimental sections.

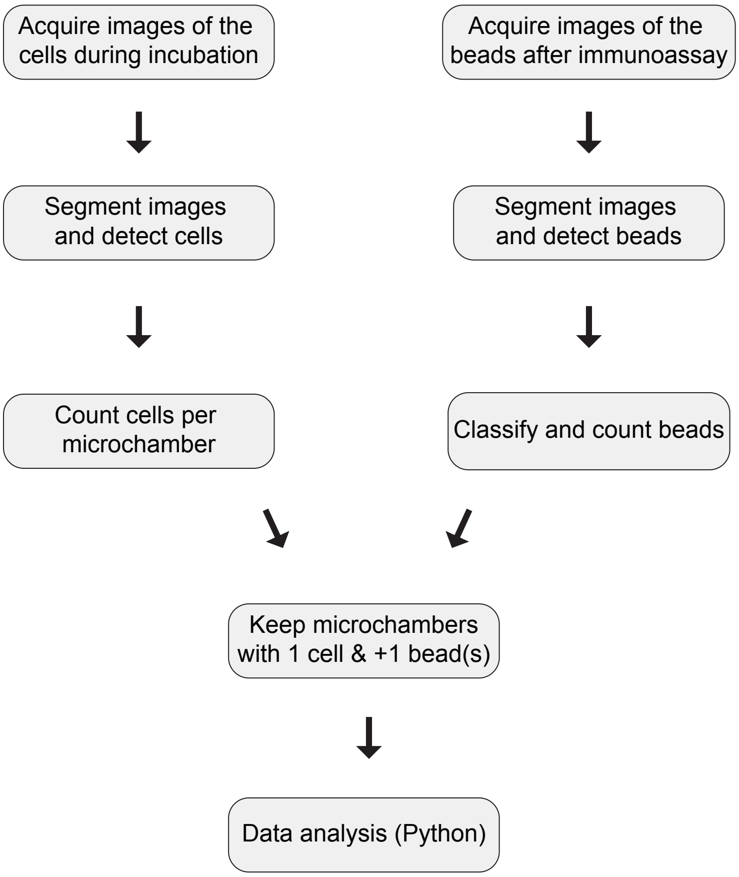

Figure S1. Image acquisition and processing pipeline of the on-chip single-cell experiments. Cells were imaged during the incubation to assess viability and obtain cell count in each microchamber. Fluorescently labelled magnetic beads were imaged after the sandwich immunoassay was completed. First, images were segmented, and a threshold-based detection of the cells and beads was achieved. After counting the number of cells and beads per microchamber and classifying the different bead types (based on their two fluorescent barcodes), the microchambers which contained a single cell in presence of one or more beads were further kept in the analysis. Raw fluorescent signals on the beads surface were background subtracted.

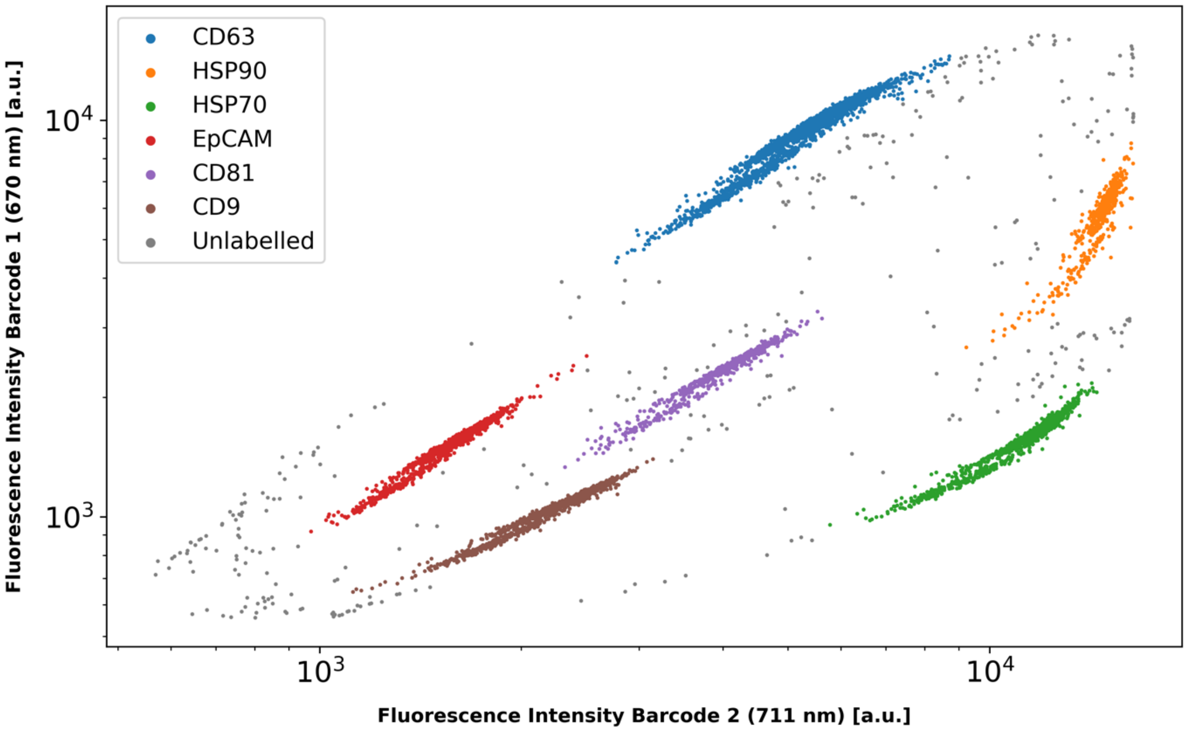

Figure S2. Classification of the different bead types based on two fluorescent barcodes. The beads functionalised with a specific EV capture antibody are inherently labelled with a specific ratio of two far-red fluorophores. Barcode 1 and barcode 2 have emission maxima around 670 nm and 711 nm, respectively. Here, magnetic beads functionalised with six different EV capture antibodies were trapped inside the microfluidic device and imaged. The six different bead types can be clearly identified and classified. Unlabelled beads may be due to beads that were out of the focusing plane.

Bead-antibody kit	Catalogue number
CD9	EPX01A-12434-901
CD63	EPX010-12433-901
CD81	EPX010-12432-901
HSP90 (alpha)	EPX010-12423-901
HSP70	EPX010-12087-901
EpCAM	EPX010-12431-901

Table S1. Catalogue numbers of the capture (bead-bound) and detection (in solution) antibody kits (ProcartaPlex™). The compatibility of the six EV markers investigated in the current study were validated with the panel configurator of the supplier.

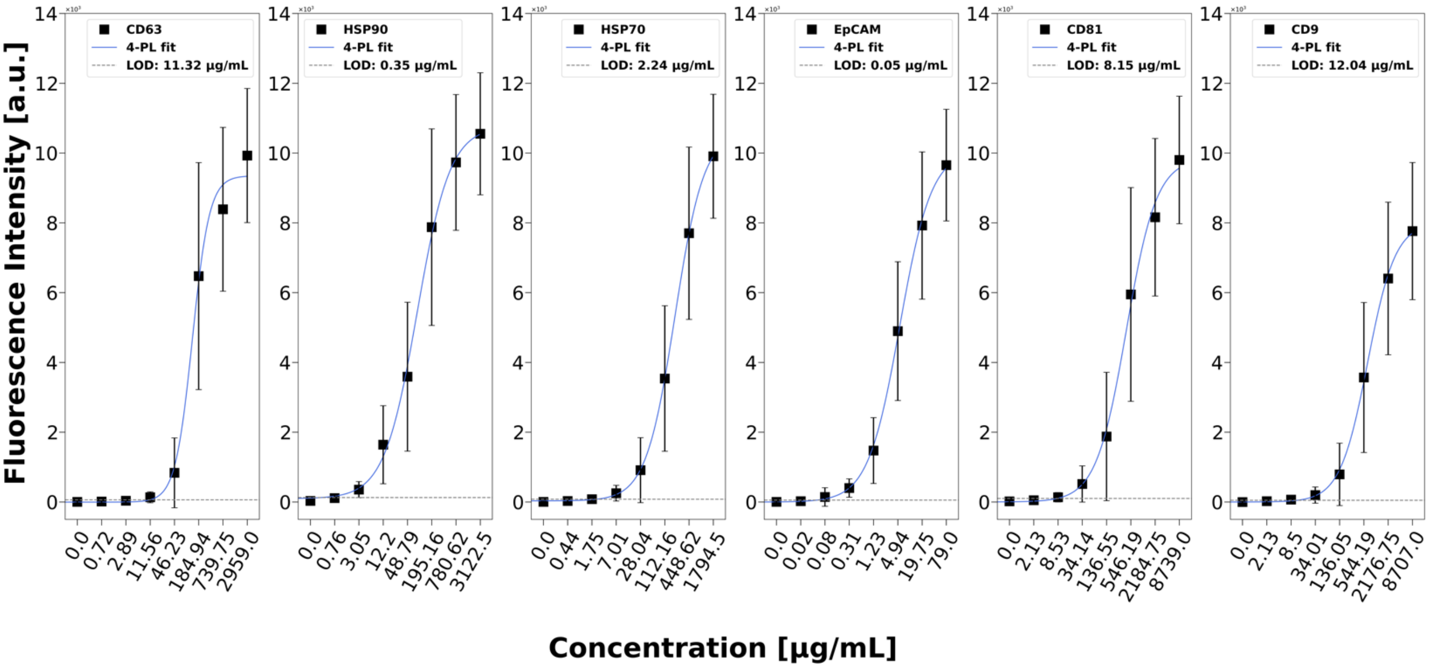

Figure S3. Calibration curves obtained on a single device for the six protein standards of interest: CD63 (LOD: 11.32 µg/mL), HSP90 (LOD: 0.35 µg/mL), HSP70 (LOD: 2.24 µg/mL), EpCAM (LOD: 0.05 µg/mL), CD81 (LOD: 8.15 µg/mL) and CD9 (LOD: 12.04 µg/mL). A 4-fold serial dilution of soluble protein standards was achieved to measure 8 distinct concentrations on a single device.

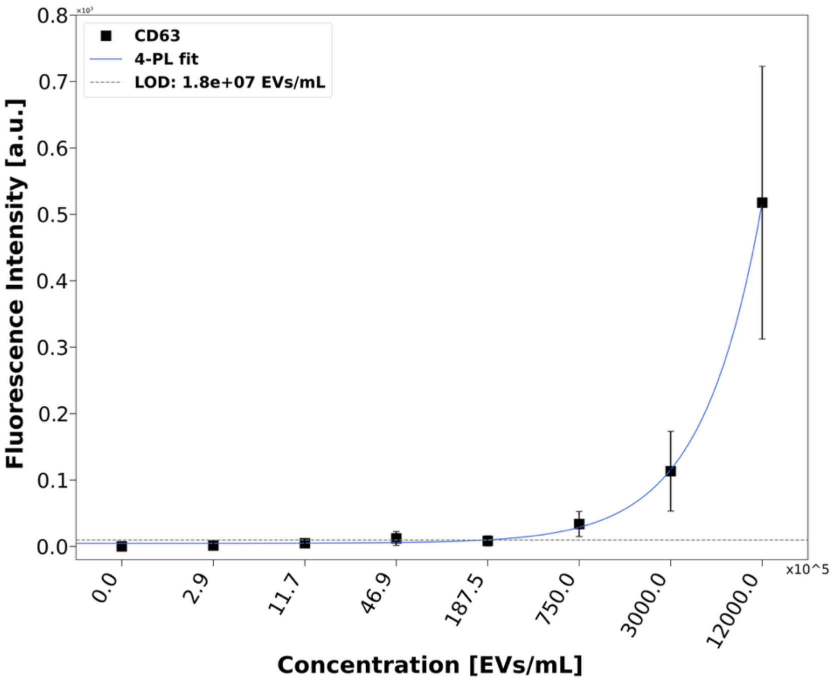

Figure S4. Calibration curve of enriched EV samples obtained on a single device. Cell culture supernatant from MDA-MB-231 cells was collected and EVs enriched by differential ultracentrifugation. A 4-fold serial dilution was loaded on-chip, EVs were captured on CD63-coated magnetic beads and detected with a mixture of detection antibodies (CD9/CD63/CD81, HSP70/HSP90). A limit of detection (LOD) of $1.8*10^7$ EVs/mL was achieved.

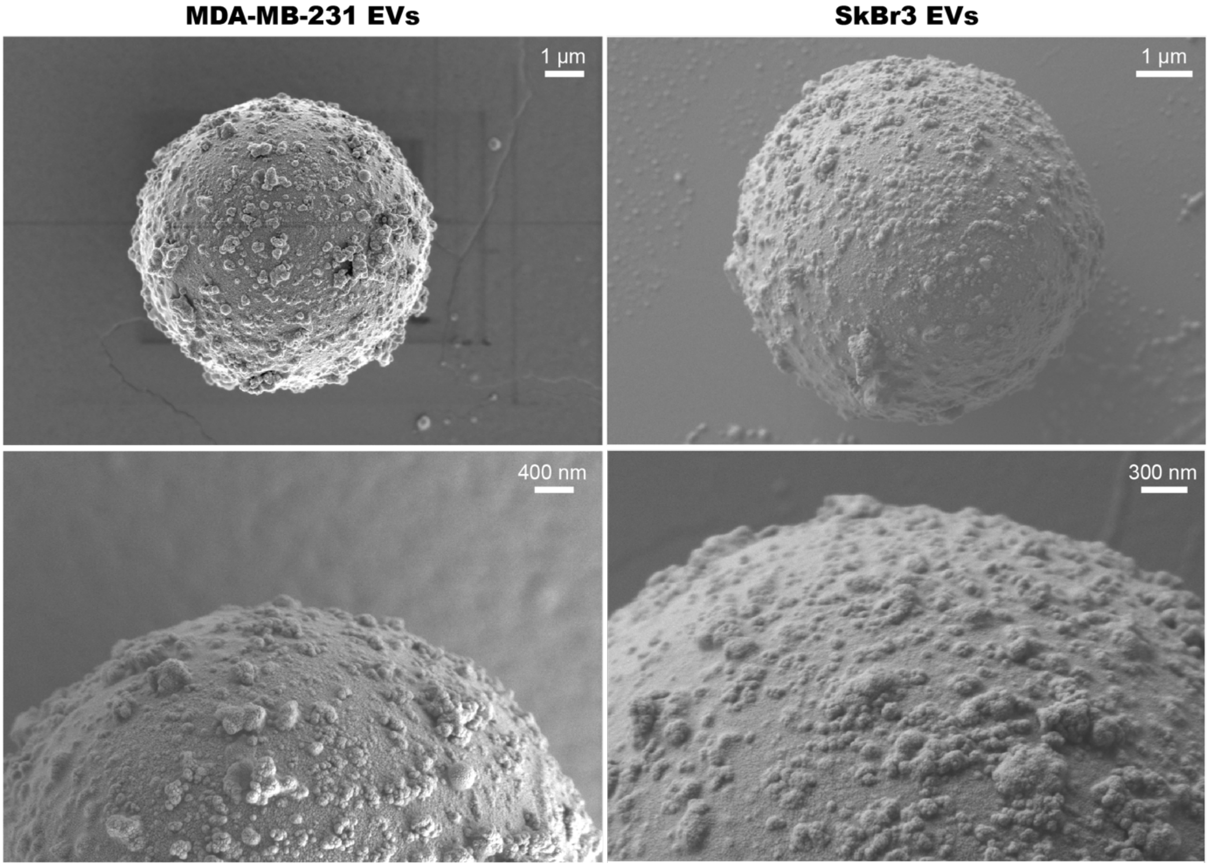

Figure S5. Scanning electron microscopy (SEM) image of bead-bound EVs. Enriched EV samples from MDA-MB-231 and SkBr3 cell culture media were captured on magnetic beads functionalised with anti-CD63 capture antibodies and imaged with SEM.

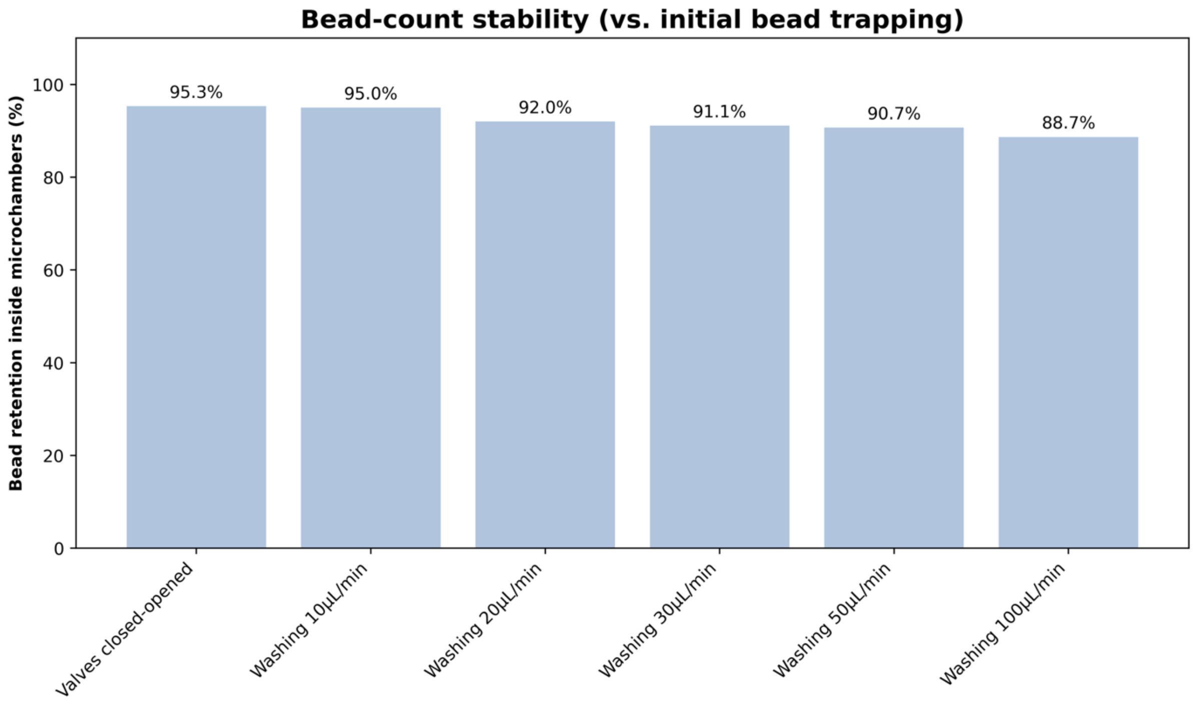

Figure S6. Bead stability inside the microchambers after the different steps of device operation. Bead retention was assessed after closing-opening the pneumatic valves, as well as after washing the channels at increasing flow rates. Right after the capturing of the magnetic beads, weakly bound beads were washed at 10 µL/min, and flow rates never exceeded 30µL/min during the experiment.

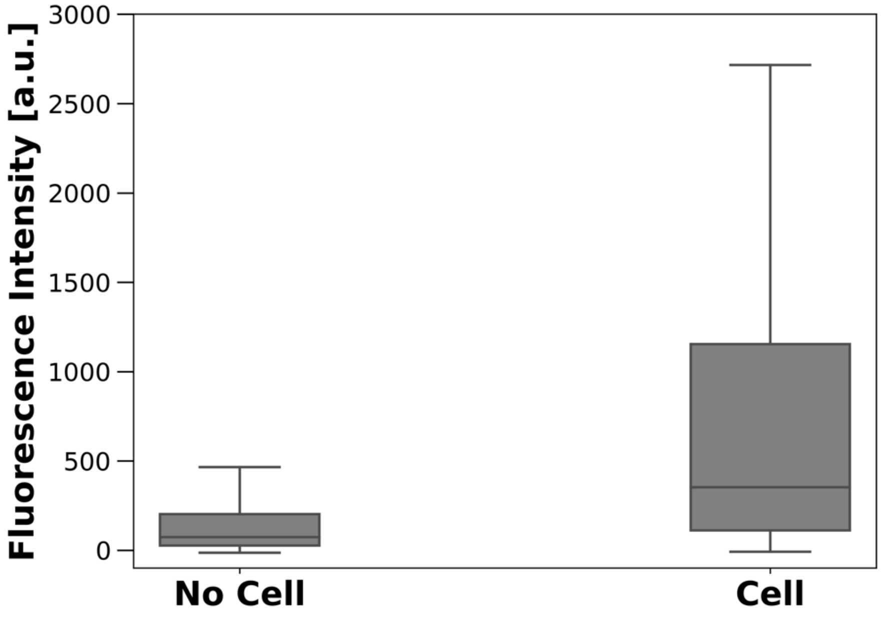

Figure S7. Comparison of the output PE fluorescent signal on magnetic bead localised in chambers which contained no cell with microchambers which contained one or more cell(s).

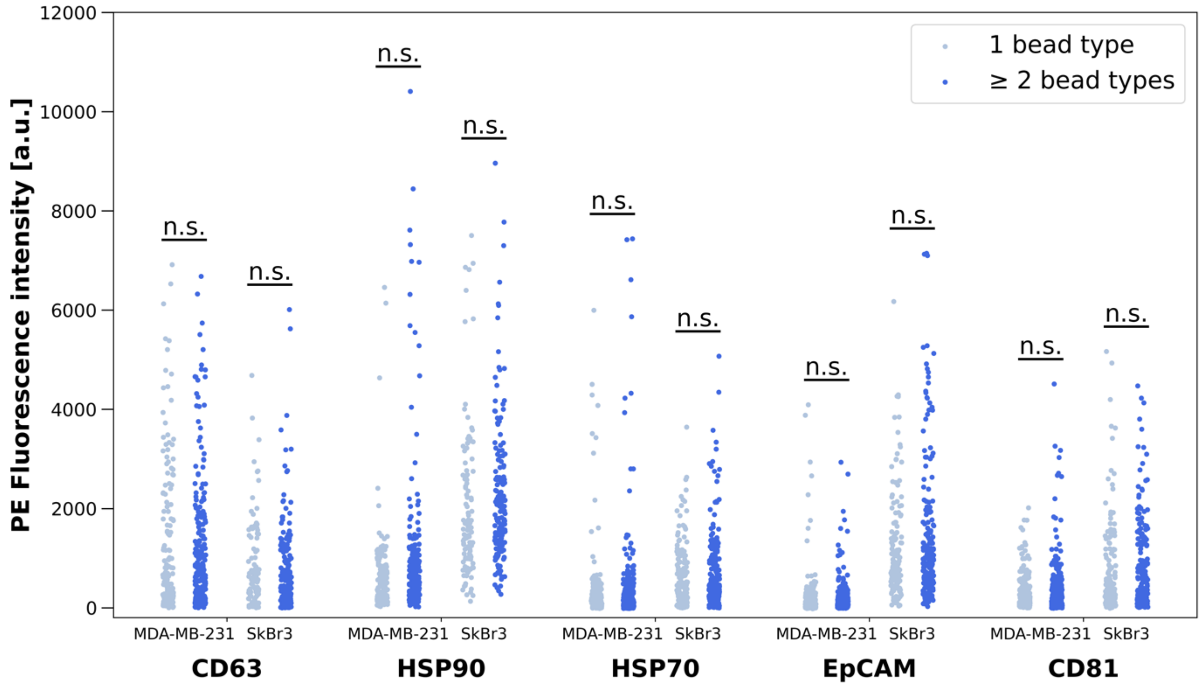

Figure S8. Comparing fluorescent signals in microchambers which contained a single bead with microchambers which contained two or more beads. The potential effect of affinity competition between different capture antibodies and the secreted EVs needed to be investigated. Indeed, the presence of several EV capture beads within the same microchamber may directly influence the binding dynamics of EVs to the functionalised beads and ultimately affect the output signal. When comparing the fluorescent signal inside microchambers which contained a single EV capture bead with those which hosted two or more bead types, no significant difference was observed for all five markers of interest. Results are shown for each cell type of interest (MDA-MB-231 and SkBr3) and for each EV membrane marker separately.

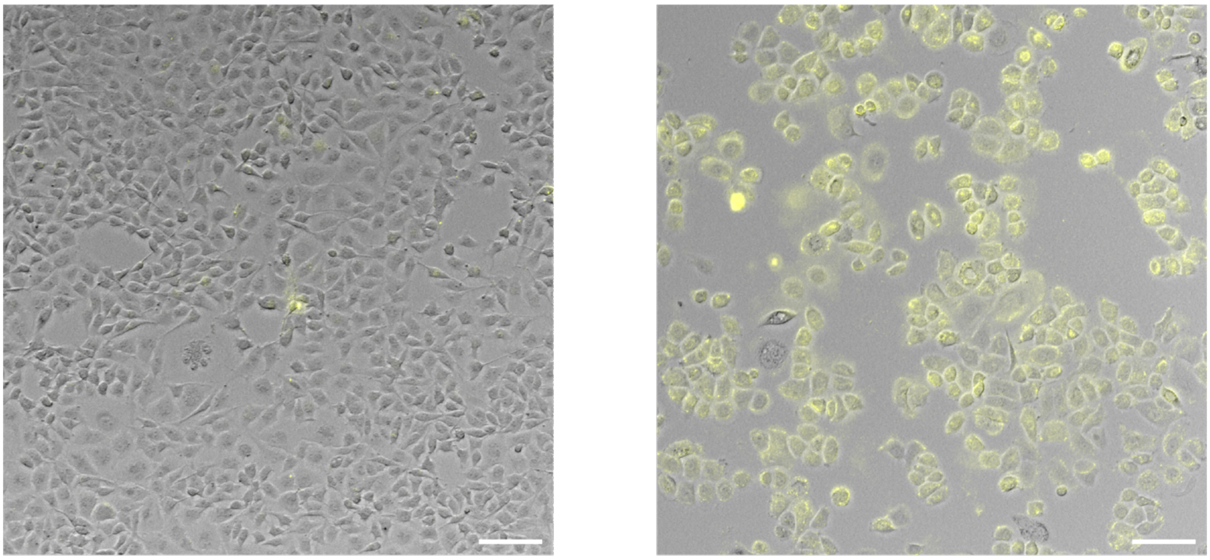

Figure S9. Brightfield images of MDA-MB-231 and SkBr3 cells in 2D cell culture. Cell membrane was labelled with antibodies against EpCAM protein (fluorescent image shown with the yellow overlay). Scale bar = 200 µm.

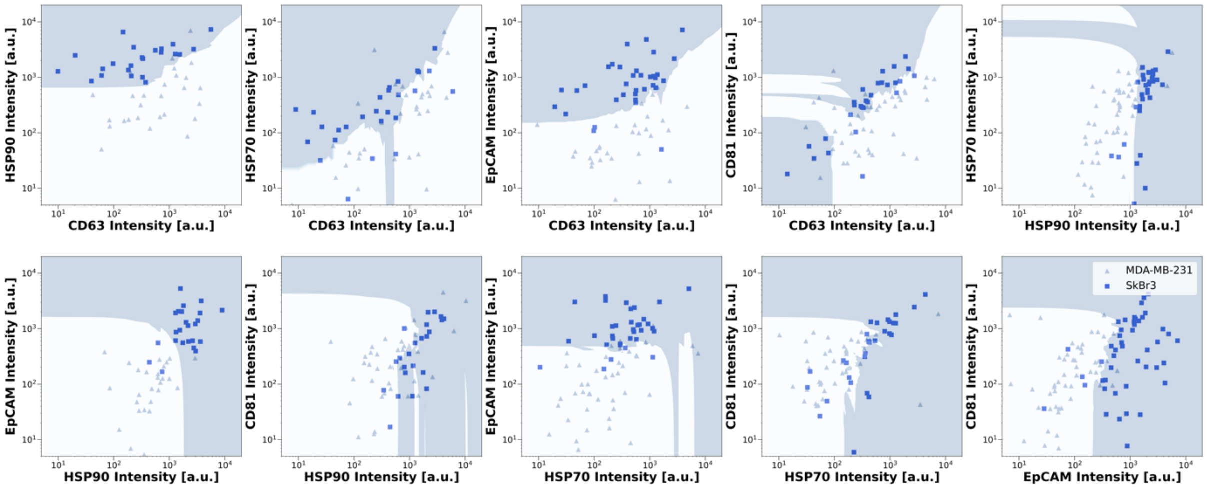

Figure S10. Complete pairwise plots for all ten EV marker pairs. The microchambers which contained two different EV capture beads were considered here. The fluorescence intensity of one EV marker is plotted with reference to the fluorescence intensity of the second EV marker. Decision boundaries obtained with the KNN classifier are displayed. For each bead pair and each cell line, $n \geq 15$ (N = 2).

EV Marker Pair	Accuracy Score (%)
CD63-HSP90	94%
CD63-HSP70	79%
CD63-EpCAM	94%
CD63-CD81	80%
HSP90-HSP70	92%
HSP90-EpCAM	93%
HSP90-CD81	77%
HSP70-EpCAM	84%
HSP70-CD81	73%
EpCAM-CD81	91%

Table S2. Accuracy score of the KNN classifier for each EV marker pair.

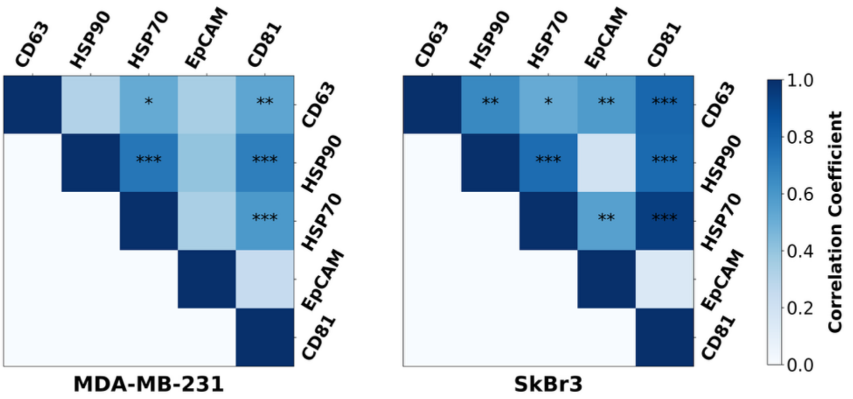

Figure S11. Correlation maps for all ten EV marker pairs for the two cell lines of interest (MDA-MB-231 and SkBr3).

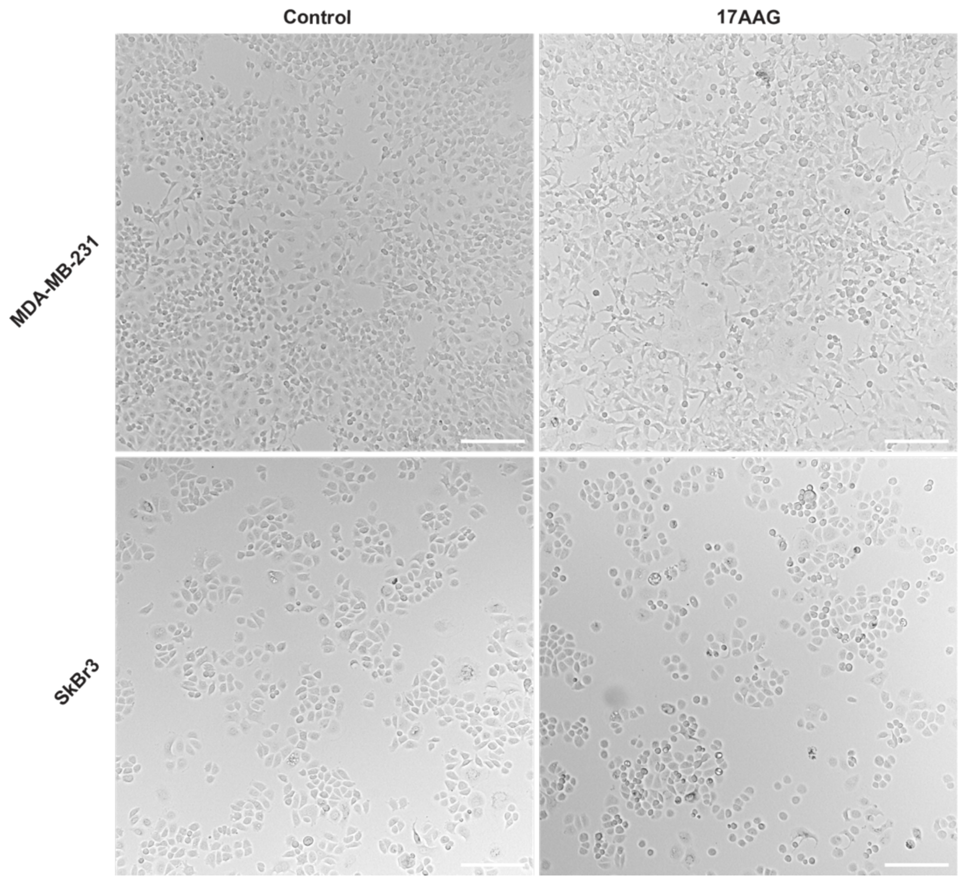

Figure S12. Brightfield images of MDA-MB-231 and SkBr3 cells during the drug treatment assay. Control (left) and 17AAG-treated (right) cells were cultured for 24 hours with DMSO or 10 µM 17AAG prior to loading on the microfluidic device. Scale bar = 200 µm.

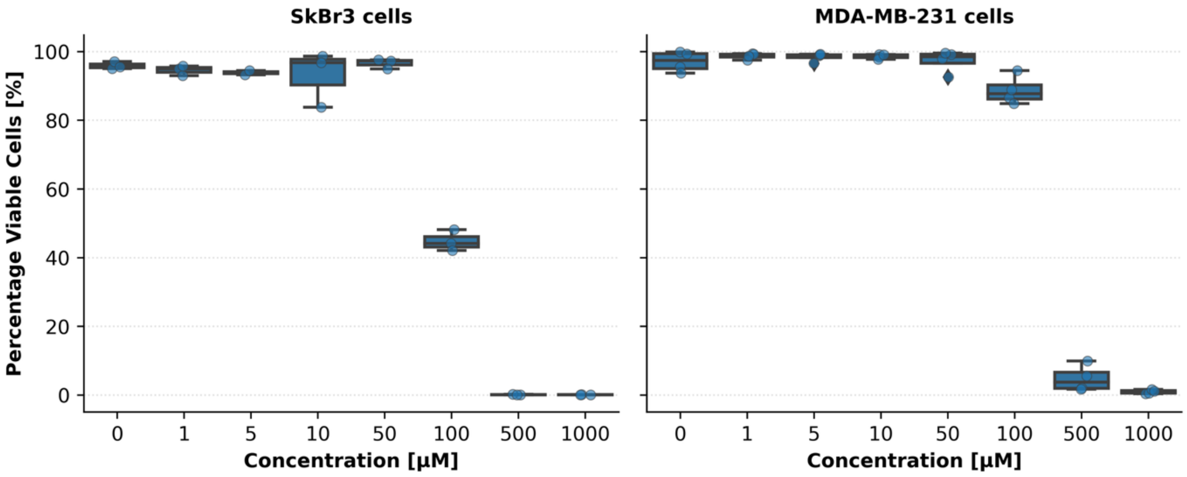

Figure S13. Cell viability assay upon treatment with different 17AAG concentrations. SkBr3 and MDA-MB-231 cells were cultured for 24h at varying drug concentrations ranging from 0-1000 µM. Viability was assessed with a Live-Dead staining. Cell viability remained above 80% until 50 µM 17AAG for both cell types.

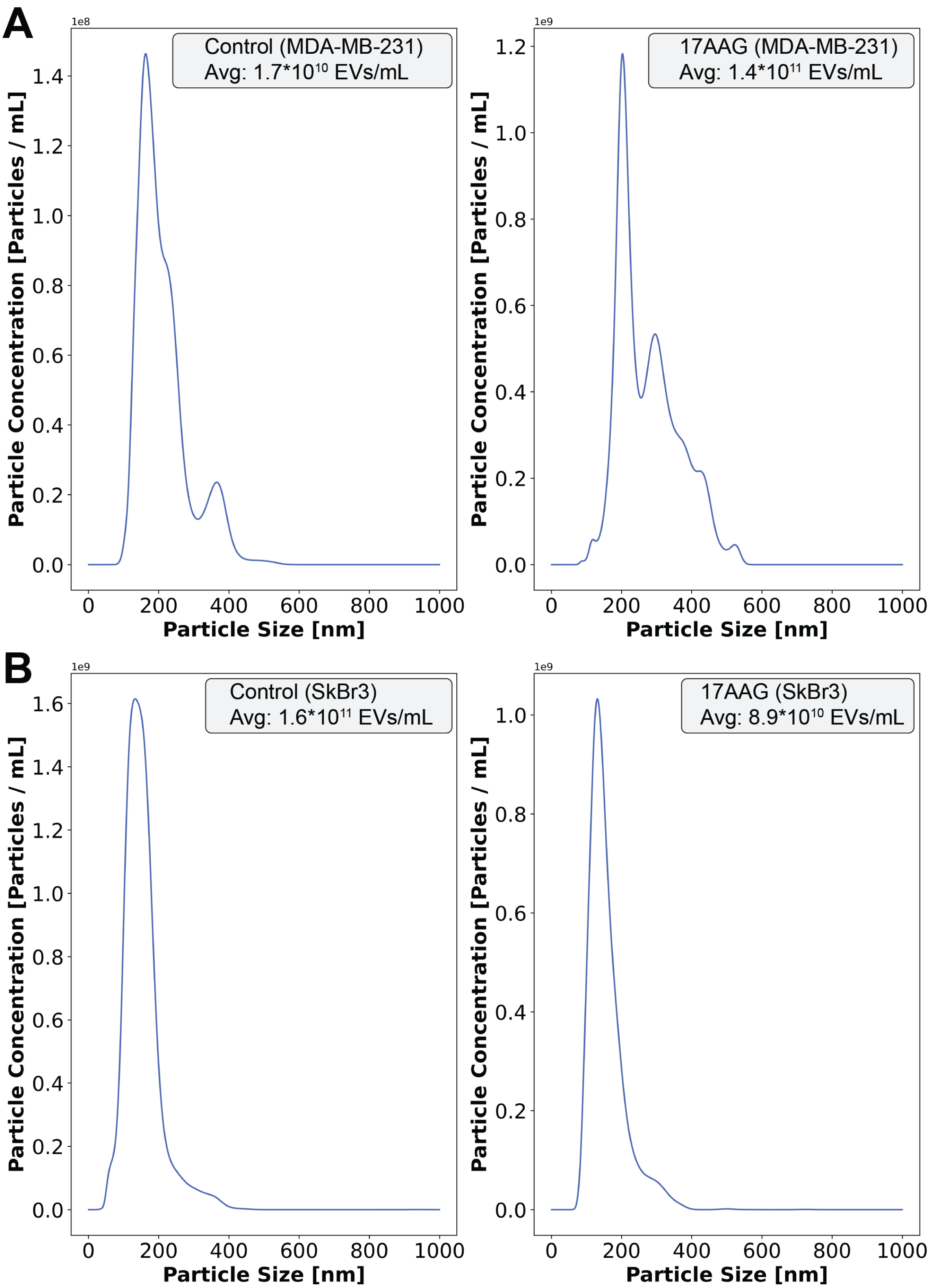

Figure S14. Nanoparticle Tracking Analysis (NTA) results of EV samples enriched from bulk cell culture media. A) MDA-MB-231 and B) SkBr3 cells were cultured for 48 hours (both in drug-treated and control conditions). Cell culture supernatant was then collected and EVs enriched by differential ultracentrifugation following a standard protocol[1]. We observe a 10-fold increase in EV concentration for MDA-MB-231 cells, while no increase in secreted EVs is measured for SkBr3 cells after drug treatment.

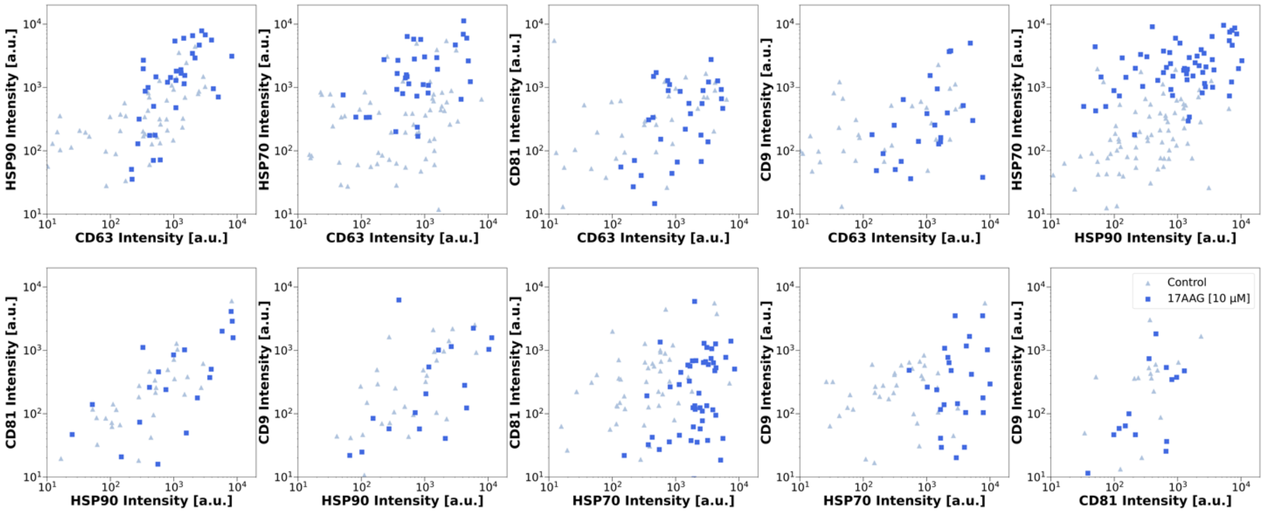

Figure S15. Complete pairwise plots for all ten EV marker pairs for the 17AAG drug treatment experiment with MDA-MB-231 cells. The microchambers which contained two different EV capture beads were considered here. The fluorescence intensity of one EV marker is plotted with reference to the fluorescence intensity of the second EV marker. For each bead pair and each condition, n ≥ 14 (N = 3).

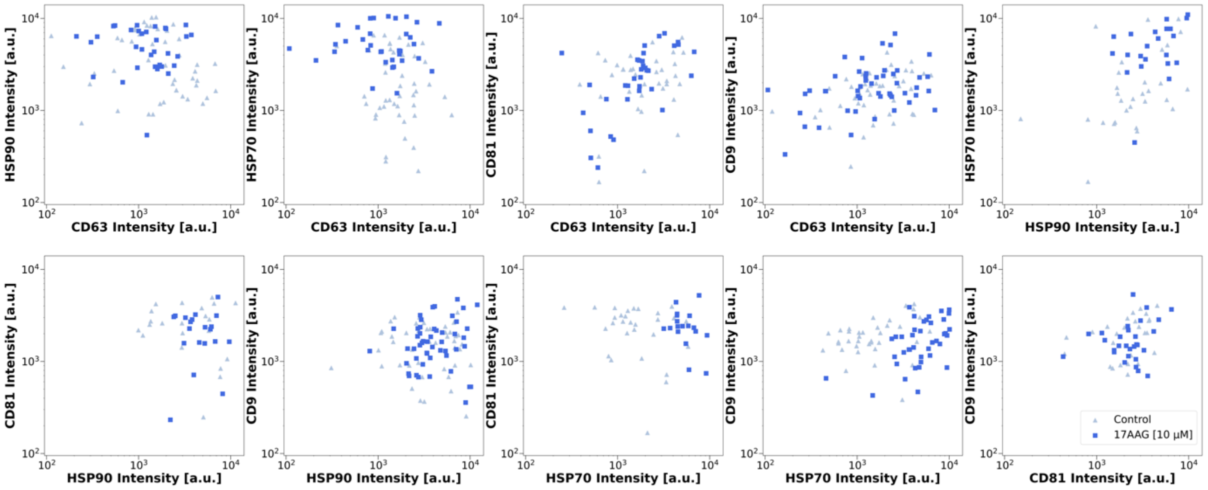

Figure S16. Complete pairwise plots for all ten EV marker pairs for the 17AAG drug treatment experiment with SkBr3 cells. The microchambers which contained two different EV capture beads were considered here. The fluorescence intensity of one EV marker is plotted with reference to the fluorescence intensity of the second EV marker. For each bead pair and each condition, n ≥ 16 (N = 3).

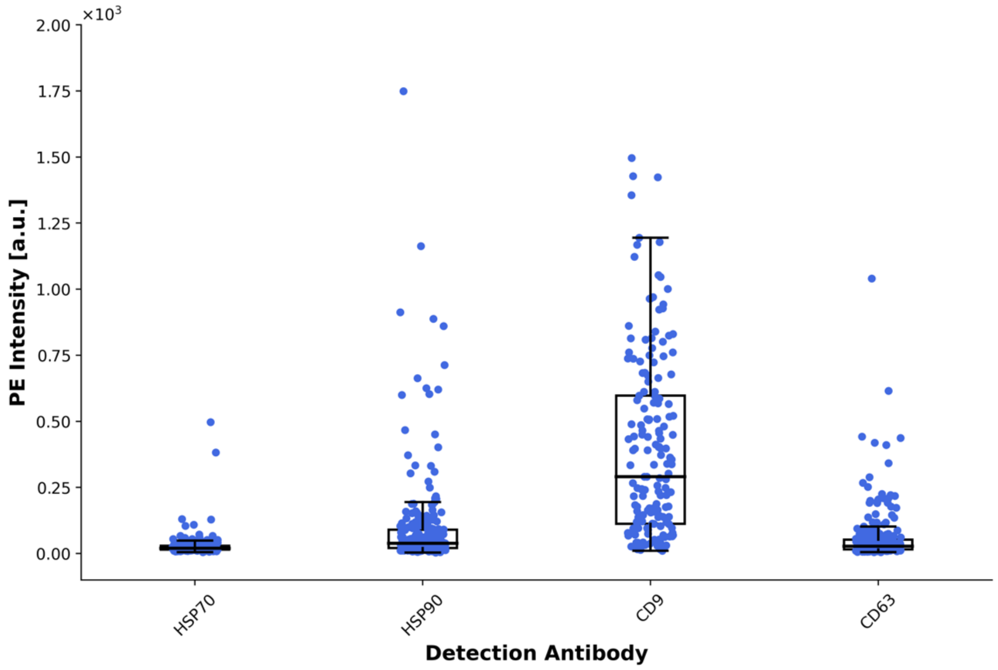

Figure S17. Colocalization of proteins on CD63-positive EVs, derived from single MCF10A cells. Cell-secreted EVs were captured on CD63-coated magnetic beads and labelled with one of four detection antibody – HSP70, HSP90, CD9 or CD63.

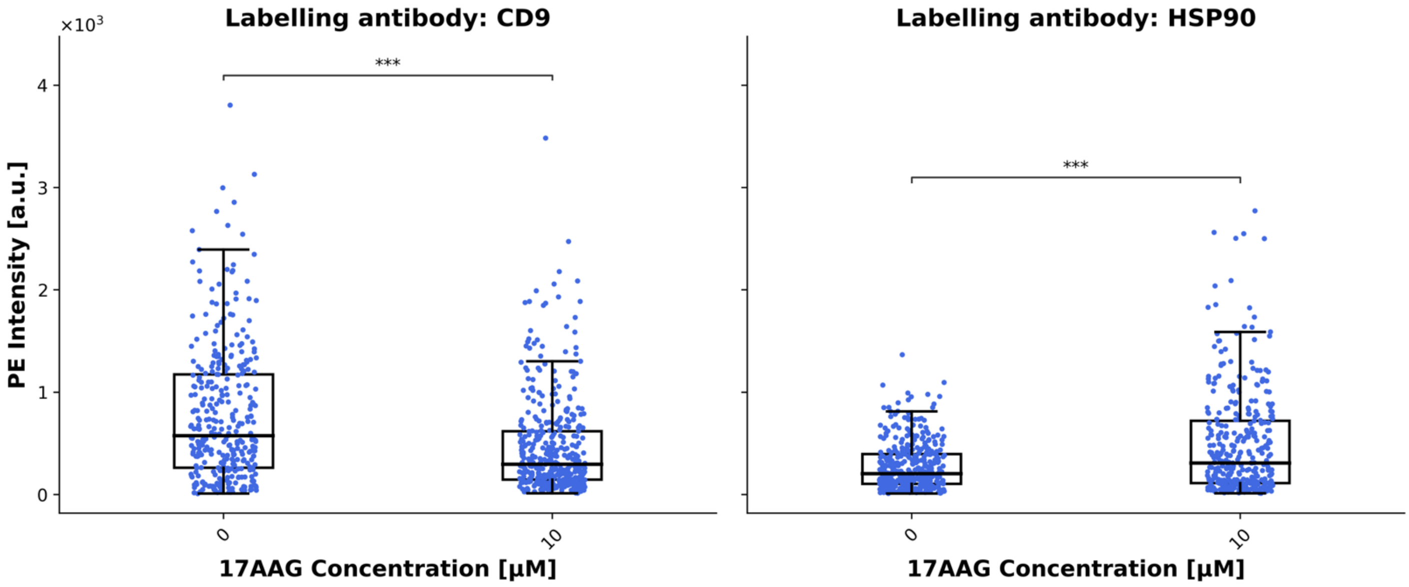

Figure S18. Effect of 17AAG on EVs secreted by healthy breast cell line MCF10A. Cells were treated with 10 µM 17AAG – and control – for 24 hours prior to loading on chip. Secreted EVs were captured on magnetic beads functionalised with anti-CD63 magnetic beads and detected with a solution of CD9 (left) or HSP90 (right) labelling antibody.

Table S3. Summary of the antibodies used for capturing (bead-bound) and detecting (in solution) the single-cell-secreted EVs in the different experimental sections.

Figure number	Types of EV capture beads	Types of detection antibodies
3	CD63 + CD81 + HSP70 + HSP90 + EpCAM	CD63 + CD81 + HSP70 + HSP90 + EpCAM (mixture)
4	CD9 + CD63 + CD81 + HSP70 + HSP90	CD9 + CD63 + CD81 + HSP70 + HSP90 (mixture)
5	CD63	CD9, CD63, CD81, EpCAM, HSP70, HSP90 (individual)

Experimental section

Microfabrication

Two distinct master moulds were fabricated by standard photolithography. The first mould for the upper layer of the device was patterned with negative structures of the hydrodynamic cell traps and the magnetic bead traps, while the second mould for the lower layer of the device was patterned with negative structures of the pressure-controlled valves. First, 2D drawings of the microstructures were designed on AutoCAD software and binary masks obtained from Selba S.A., Switzerland. Negative SU-8 photoresist (Kayaku Advanced Materials) was spin coated onto 4-inch silicon wafers (MicroChemicals WSV4 0100) and prebaked at 65˚C and 95˚C for 1 and 10 minutes, respectively. Photoresist viscosity and spin coating speed were tuned to deposit microstructures of well-defined thickness. The silicon wafer of the upper layer was first spin coated at 3000 rotations per minute (rpm) with SU-8 3025 to obtain hydrodynamic cell traps of 25 µm height. After 250 mJ exposure to ultraviolet (UV) light through the corresponding binary mask, the wafer was baked post-exposure for 1 and 3 minutes at 65˚C and 95˚C, respectively. A second layer of SU-8 3010 was then spin coated at 4000 rpm to pattern the magnetic bead traps with a 10 µm depth. The wafer was exposed to 400 mJ UV light and baked post-exposure for 1 and 3 minutes at 65˚C and 95˚C, respectively. The master mould of the lower layer consisted of a single layer of SU-8 3010 spin coated at 1100 rpm and exposed at 600 mJ, reaching a thickness of 20 µm. Identical pre- and post-exposure baking parameters were used. Both master moulds were developed in mr-Dev 600 solvent for 10 minutes and hard-baked for 30 and 120 minutes at 65˚C and 200˚C, respectively. Finally, the patterned silicon wafers were silanised (Sigma 448931-10G) overnight in a vacuum chamber.

Assembling of the microfluidic device

The two-layered polydimethylsiloxane (PDMS) devices were fabricated by soft lithography. First, PDMS (Silgard^{TM} 184 Silicone Elastomer Base) was mixed with curing agent (Silgard^{TM} 184 Silicone Elastomer Curing Agent) in a 10:1 ratio and degassed under vacuum. The polymer mix was directly poured onto the master mould of the upper layer up to a 0.5-1 cm height and cured at 80˚C for 2 hours. The lower layer of the device required a thin and controlled thickness of PDMS. Approximately 5 g of uncured polymer mix was poured at the centre of the master mould and spin coated at 2200 rpm for 60 seconds, following a curing at 80˚C for 30 minutes. A thickness of 40 µm was reached. The two PDMS layers were then aligned under an optical microscope and chemically bonded with curing agent and 1 hour baking at 80˚C. 1 mm-diameter holes were punched through the inlets and outlets of all channels using a biopsy hole puncher (Miltex 33-31AA). In addition, three holes of 1.5 mm diameter were punched on each side of the PDMS chips, which later served as anchor points for the magnet holder. After cleaning the PDMS chips and glass slides (0.16-0.19 mm thick) with scotch tape, both surfaces were activated in an oxygen plasma for 30 seconds and bonded together. The finalised chips were stored at room temperature until use.

Preparation of the microfluidic device

For the device preparation, the pressure-controlled channels were filled with diH_2O. For each inlet, an Eppendorf filled with diH_2O was closed with an airtight cap (Fluigent P-CAP). One end of the cap was connected to a pressure pump (Flow EZ^{TM}, Fluigent), and the other end directly connected to one of the pressure-controlled channel inlets. The channels were then filled with diH_2O by applying a 500-mbar pressure until all air was pressed out through the PDMS. The upper flow-controlled channels were coated with a solution of 1% w/v bovine serum albumin (BSA) in PBS using a similar principle. Eppendorf's were however filled with the BSA solution instead of diH_2O, and the channels outlets were

clogged with a pipette tip filled with cured PDMS. After 30 minutes of incubation with the BSA coating solution, the pressure pumps were disconnected from the flow-controlled channels and the outlets unclogged. Each flow-controlled channel inlet was then connected to a 1 mL glass syringe (Agilent 1560) filled with Dulbecco's Modified Eagle Medium (DMEM) and mounted on a syringe pump (Nemesys, Cetoni). All pressure and syringe pumps were connected to the microfluidic chip via transparent Tygon tubing with inner diameter 1/50 inches and outer diameter 1/16 inches (Masterflex ND-100-80).

Cell culture

Breast cancer cell lines MDA-MB-231 (ATCC HTB-26) and SkBr3 (ATCC HTB-30) were cultured in Dulbecco's Modified Eagle Medium (DMEM, Gibco 31885-023) supplemented with 10% Foetal Bovine Serum (FBS, Gibco A5256701) and 1% Penicillin-Streptomycin (Gibco 15140-122). When 80% confluency was reached, cells were washed for 5 minutes in Phosphate Buffered Saline (PBS, Gibco 10010-023), then detached in 0.25% Trypsin-EDTA (Gibco 25200-056) and passaged in a 1/5 ratio.
For the on-chip drug treatment assays, MDA-MB-231 and SkBr3 cells were treated for 24 h with 10 µM 17AAG (Sigma J66960.MB), or the control media with a corresponding volume of Dimethyl Sulfoxide (DMSO, PanReac AppliChem A3672,0100). The 17AAG compound was initially dissolved and aliquoted in DMSO at 25 µg/mL, then stored at -20 ˚C until use. Prior to on-chip single-cell measurements, cells were washed with PBS for 5 minutes, detached as described above, and centrifuged down at 500 g for 5 minutes. The cell pellet was then resuspended in DMEM containing 1 µg/mL CellTrace Calcein Violet, AM (Invitrogen C34858) and incubated for 30 minutes at 37˚C with 5% CO_2. Following 3 washes with PBS, the cells were finally resuspended in DMEM at a cell density of 0.8 million cells/mL and loaded onto the microfluidic device.

EV enrichment from cell culture media

Isolation and enrichment of EVs from MDA-MB-231 and SkBr3 cell culture media was achieved by differential ultracentrifugation, following previously established protocol[1]. Briefly, confluent cell cultures were cultured in DMEM supplemented with 10% exosome-depleted FBS (Gibco A2720801) and 1% Penicillin-Streptomycin for 48 h. The culture media was collected, centrifuged for 5 minutes at 500 g, then for 10 minutes at 2000 g and finally for 30 minutes at 20'000 g to remove cellular debris. The supernatant was collected and centrifuged for 90 minutes at 100'000 g. After discarding the supernatant, the pellet containing extracellular vesicles was resuspended in filtered PBS and stored at -20˚C until use. The concentration and size distribution of enriched EVs was quantified using Nanoparticle Tracking Analysis (NTA, NanoSight NS300).

Scanning electron microscopy (SEM) of bead-bound EVs

Bead-bound EVs were measured with SEM (Zeiss, Crossbeam 550). Samples preparation was adapted from a previously established protocol[2]. First, EV samples were enriched from cell culture media of MDA-MB-231 and SkBr3 cells (as described above). EVs samples were incubated with magnetic beads functionalised with anti-CD63 capture antibodies for 2 hours at room temperature, on a shaker set at 150 rpm. The beads were washed three times with a HEPES buffer solution (pH ~ 7-7.5). Then, bead-bound EVs were fixed with 2% paraformaldehyde (PFA) in HEPES buffer for 30 minutes at room temperature. The beads were washed three times with HEPES buffer and sequentially washed in ethanol at increasing concentrations (30%, 50%, 70%, 90% and 100%), with five minutes incubation for each step.

The beads resuspended in 100% ethanol were loaded and dried in ambiant air on a blank silicon wafer. Last, the samples were gold sputtered for improving SEM signal.

References

(1) Konoshenko, M. Yu.; Lekchnov, E. A.; Vlassov, A. V.; Laktionov, P. P. Isolation of Extracellular Vesicles: General Methodologies and Latest Trends. *Biomed Res Int* **2018**, *2018*, 1–27. https://doi.org/10.1155/2018/8545347.

(2) Collier, M. E. W.; Allcock, N.; Sylvius, N.; Cassidy, J.; Giorgini, F. Examination of the Enrichment of Neuronal Extracellular Vesicles from Cell Conditioned Media and Human Plasma Using an Anti-NCAM Immunocapture Bead Approach. *BMC Methods* **2025**, *2* (1), 12. https://doi.org/10.1186/s44330-025-00034-7.
